# Metals, cardiovascular risk, and the interplay with oxidative stress: a mini-review

**DOI:** 10.1590/1414-431X2025e14466

**Published:** 2025-06-20

**Authors:** J.G.P. Pires, I.R.G. Schereider, F.W.S. Cibin, F.A. Scorza, G.A. Wiggers, D.V. Vassallo

**Affiliations:** 1 Curso de Medicina, Escola Superior de Ciências da Santa Casa de Misericórdia de Vitória - EMESCAM, Vitória, ES, Brasil; 2 Departamento de Ciências Fisiológicas, Centro de Ciências da Saúde, Universidade Federal do Espírito Santo, Vitória, ES, Brasil; 3 Programa de Pós-Graduação em Bioquímica, Universidade Federal do Pampa, Uruguaiana, RS, Brasil; 4 Disciplina de Neurociência, Departamento de Neurologia e Neurocirurgia, Universidade Federal de São Paulo/Escola Paulista de Medicina (UNIFESP/EPM), São Paulo, SP, Brasil; 5 Programa de Pós-Graduação Multicêntrico em Ciências Fisiológicas, Universidade Federal do Pampa, Uruguaiana, RS, Brasil

**Keywords:** Oxidative stress, Free radical, Redox, Heavy metal, Cardiovascular system, Antioxidant agents

## Abstract

Oxidative stress plays a key role in the mechanisms underlying pathophysiological processes, such as inflammation, age-related degenerative phenomena, atherosclerosis, hypertension, cancer, diabetes mellitus, neurodegenerative diseases, xenobiotic toxicity, among others. It is generated by the production of free radicals, resulting from the oxidative metabolism of cells. Oxidative stress is an important defense against infections. It acts specifically as a vasodilator and helps modulate antioxidant mechanisms. However, the effects become harmful when its production increases or antioxidant mechanisms are excessively reduced. Toxic metals from environmental and occupational exposure are silent agents that induce oxidative stress. Metals such as mercury (Hg), aluminum (Al), cadmium (Cd), and lead (Pb) are known to be toxic to various organs and tissues in our body. The present mini-review focuses on the cardiovascular system, considering that the interplay between oxidative stress and toxic metals acting silently is involved in their harmful effects, especially on the etiopathogenesis of cardiovascular disorders. A brief review is also given regarding the mechanisms of modulation of redox homeostasis by organic mechanisms, pharmacological approaches that can act directly or indirectly as antioxidants, and food-derived compounds that appear to be effective inhibitors of oxidative stress, thus preventing the harmful effects of free radicals.

## Introduction

The concept of oxidative stress was first formulated by Sies et al. at a scientific conference in 1985 (1). Since then, it has expanded to now cover practically all organs and systems. Oxidative stress is involved in various pathophysiological processes such as inflammation, aging, atherosclerosis, hypertension, cancer, diabetes mellitus, neurodegenerative diseases, mental disorders, xenobiotic toxicity, preeclampsia/eclampsia, age-related macular degeneration, among others. The concept was later adapted by Jones (2), who suggests that oxidative is “a disruption of redox signaling and control”. Regarding the cardiovascular system, oxidative stress is somehow involved in the etiopathogenesis of arterial hypertension, coronary artery disease, heart failure, and other conditions (1-6).

In recent decades, environmental and occupational exposure to heavy metals has been considered a risk factor for cardiovascular diseases (CVDs) (7). Several studies have been carried out evaluating the cardiotoxic effects of exposure to various metals normally found in the environment or occupational settings (8). Additionally, the *in vitro* and *ex vivo* mechanisms of action of nonessential metals such as mercury (Hg), cadmium (Cd), lead (Pb), and aluminum (Al) on the cardiovascular system have been explored (9-13). Importantly, these studies indicate that oxidative stress is one of the main mechanisms involved in the metal-induced damage to the cardiovascular system. This appears to be related to the ability of toxic metals to replace essential divalent cations in several enzymes and metalloproteins, impairing vascular function, inducing oxidative stress, and consequently leading to cardiovascular events such as hypertension and atherosclerosis (14). This mini-review addresses the above aspects and the worrying and still little-known effects of metal exposure.

## Basic principles of chemistry

The bonds between the atoms of a molecule or chemical species involve the mobilization of electrons, in particular the electrons of the outermost electronic layer, called the valence layer. The atomic nuclei (where protons and neutrons are located) do not have direct participation in these reactions. Electron mobility can be complete, as in the ionic bonds common in inorganic salts, or it can involve the sharing of electrons in the various types of covalent bonds. In both cases, parameters or properties can be attributed to each chemical element, such as atomic radius, chemical reactivity, electronegativity, electropositivity, oxidation number (Nox), among others. Elements, when combined, usually have more than one Nox; for example, Fe has +2 (ferrous) and +3 (ferric) Nox. In simple substances, such as O_2_ and N_2_, the oxidation number of the element is zero (15,16).

Most chemical reactions, including those that occur in living organisms, involve changing the oxidation number of the atom or chemical species. In these reactions, called oxidation and reduction reactions (or, in short, oxidation-reduction, or redox reactions), the atom that gains electrons undergoes reduction (that is, reduction of its Nox), while the atom that loses electrons undergoes oxidation. The name oxidation probably derives from the fact that in the past it was considered that any reaction of this type only occurred in the presence of molecular oxygen (O_2_). The oxidizing or reducing agents are not the isolated atoms, but the substance or chemical species that contain them (16).

Electron gains and losses are known as complete in ionic bonds and incomplete in covalent bonds, where the electron pair tends to get closer to the atom with the lowest Nox. Simply put, to quantify the affinity for electrons, another characteristic of the chemical element was defined, called electronegativity. In the periodic table, the most electronegative elements are F, O, and N in descending order. For example, in the water molecule, we have two covalent bonds involving H and O, and the electron cloud that defines the covalent bond is closer to O, which is the most electronegative element of the two. In this case, the Nox of H is +1 and the Nox of O is -2. As oxygen (O) is one of the most electronegative elements, its introduction into a chemical species was called oxidation. On the other hand, the introduction of H into a chemical molecule or species causes its reduction. In short, and using a broader definition, the chemical reaction of oxidation is the loss of electrons to a chemical species, while reduction is the gain of electrons from a chemical species, with a consequent decrease in its Nox. In summary, life as we know it is a complex set of redox reactions, and redox homeostasis along with pH control mechanisms is fundamental to our health (1).

O_2_ is essential for the survival of all aerobic organisms. Aerobic energy production is dependent on mitochondrial oxidative phosphorylation (1). In this process, O_2_ acts as the final electron acceptor for cytochrome-c oxidase, which catalyzes the reduction of O_2_ to H_2_O. However, during this event, partially reduced and highly reactive O_2_ metabolites may be formed. Superoxide anion (O_2_^•-^) and hydrogen peroxide (H_2_O_2_) are O_2_ metabolites formed by one- and two-electron reductions of O_2_, respectively. In the presence of transition metal ions, the even more reactive hydroxyl radical (OH^•^) can be formed through the Fenton reaction. These partially reduced O_2_ metabolites are often referred to as “reactive oxygen species” (ROS) due to their high reactivity.

## Origins of the oxidative stress concept

As mentioned previously, ROS are by-products of cellular metabolism, primarily in mitochondria due to their role in the respiratory chain (1). As such, mitochondria are critically involved in the maintenance of cellular homeostasis, and altered mitochondrial functions have been linked to several pathologies (17). Other endogenous sources of ROS include enzymes such as NADPH-oxidase (NOX enzymes), xanthine oxidase, and myeloperoxidase (in macrophages).

Among the physiological functions of ROS are phagocytosis, energy production, cell growth regulation, intercellular signaling, and synthesis of important biological substances. On the other hand, physiological levels of molecular ROS, such as H_2_O_2_, act as second messengers in the cell signaling process and modulate various intracellular proteins (18). In general, the moderate production of these highly reactive chemical species may initially represent a type of “metabolic defense” of the tissues, a physiological “oxidative eustress” (as opposed to “oxidative distress”) (18). It can later, however, exert several deleterious effects. Oxidative stress causes significant cellular changes and is characterized by an intense production of ROS, which are usually neutralized by antioxidants (15,16). Thus, when the protective action of endogenous antioxidant mechanisms fail and/or the endogenous production of free radicals and similar chemical species exceeds a certain threshold, a process of oxidative stress occurs.

This concept was initially proposed by H. Sies in the 1980s and was accepted by the biomedical sciences (1,18), despite occasional criticisms (19). Currently, as markers of oxidative stress are needed for clinical use, new concepts have emerged, such as the evaluation of GSH/GSSH and Cys/Cyss interactions, instead of the isolated measure of antioxidants and pro-oxidants (2). The isolated analysis of antioxidants and pro-oxidants has been traditionally used to measure oxidative stress. However, the implementation of this approach as a reliable clinical marker of oxidative stress is challenging. Therefore, the balance between pro-oxidants and antioxidants through the interaction of GSH/GSSH and Cys/CySS redox state in plasma has been successfully used (2).

The excess of ROS and similar chemical species, such as nitric oxide (NO) and peroxynitrite (ONOO-), can cause several types of biological damage, including lipid peroxidation of biological membranes (plasmalemma and membranous organelles such as the endoplasmic reticulum, mitochondria, complex of Golgi, and lysosomes) and modifications in cellular macromolecules (enzymes, other proteins, DNA, and RNA) (19). In this regard, the hydroxyl free radical is generally the most reactive and therefore potentially the most harmful (1). The stimuli capable of generating such highly reactive chemical species can be physiological or pathological in nature (e.g., inflammation, infections, xenobiotic intoxication, exposure to toxic metals) and are still not fully understood. In addition, ROS can cause many biochemical injuries at different sites, including the central nervous system and the cardiovascular system (7,8,19-24).

To avoid oxidative stress, cells and tissues have enzymatic and non-enzymatic mechanisms capable of inhibiting this pathological effect resulting from excess ROS. As an example of a protective enzymatic mechanism, superoxide dismutase (SOD) forms the first line of defense against oxidative stress, being the body's greatest defense system against O_2_^•-^ by catalyzing the dismutation of O_2_^•-^ into O_2_ and H_2_O_2_. In turn, the H_2_O_2_ produced by the dismutation of O_2_^•-^ by SOD is converted into water and O_2_ by catalase, an intracellular antioxidant enzyme typically found in peroxisomes (23). Moreover, glutathione peroxidase (GPx) is a cytosolic enzyme that also catalyzes the reduction of H_2_O_2_ to H_2_O and O_2_ and the reduction of peroxide radicals to alcohol and oxygen. The level of glutathione (GSH), one of the most important endogenous non-enzymatic antioxidants, is believed to be a limiting factor in this process, which requires the maintenance of a high GSH/GSSG ratio achieved by the action of glutathione reductase (GR). GSH also serves as a cofactor for various other enzymes, such as glutaredoxin (GRXs) and glutathione-S-transferase (GST), which play crucial roles in cell detoxification. In fact, the GSH system (GSH, GSH-derived metabolites, and GSH-dependent enzymes) is one of the important endogenous antioxidant systems, which can maintain cellular redox balance and prevent oxidative damage and cell death (2,25). Other non-enzymatic mechanisms include the different antioxidant vitamins, in particular vitamins C and E (1,2). Moreover, several compounds with antioxidant potential, including nutraceuticals, have been studied and considered as a therapeutic approach to pathological alterations involving oxidative stress (26).

## Oxidative stress and the cardiovascular system: general aspects

An adequate balance between the generation and inactivation of ROS is necessary because these species modulate vascular function, either by direct action caused by oxidative stress or by activating intracellular signaling pathways that cause cell proliferation, vascular remodeling, development of an inflammatory process, and alteration in vascular tone (3).

O_2_^•-^ is an agent with high oxidizing power found in the vascular system. It is produced by enzymes such as NOX and xanthine oxidase (which uses xanthine and hypoxanthine). In addition to its effects on different vessels and organs, O_2_^•-^ can generate another ROS (ONOO-) when reacting with NO (27). By the action of the enzyme SOD, O_2_^•-^ is converted into H_2_O_2_, which through GPx, catalase, and peroxiredoxins (Prxs), is converted into water (6). These systems are essential for the detoxification of H_2_O_2_ and ONOO- and are the major factors responsible for the regulation of redox signaling in the cardiovascular system. Excess ROS can activate signaling pathways that promote smooth muscle contraction and growth, and vascular inflammation. H_2_O_2_ can also be metabolized by myeloperoxidase to form hypochlorous acid (HOCl) or react with transition metals to form OH^•^, the most reactive ROS (28,29).

The NOX family of NADPH oxidase is expressed in various tissues involved in biological functions such as defense and cell growth (30). Also, the three nitric oxide synthase (NOS) isoforms can generate O_2_^•-^ when they are uncoupled. For example, endothelial nitric oxide synthase (eNOS) uncoupling may occur due to the absence of the cofactors L-arginine and tetrahydrobiopterin (BH4), or by the action of ONOO- (31,32).

Genetic and epigenetic factors together with non-genetic factors, such as age, hormonal profile, diet, and regular physical activity, are known to participate in the etiopathogenesis of arterial hypertension. It is postulated that angiotensin II produced in tissues is largely responsible for the increase in ROS expression, which causes oxidative stress (27). The presence of oxidative stress was also detected in the bulbar areas of the sympathetic control in several animal models of arterial hypertension, particularly in experimental renovascular hypertension (33,34).

Atherosclerosis is the most prevalent underlying condition in most cardiovascular diseases and syndromes. Endothelial dysfunction plays a key role in the initiation and progression of many of these pathological conditions, and oxidative stress is a major contributor to endothelial dysfunction (24). In the 1980s, pioneering experiments in rabbit aorta had already shown that endothelium-dependent vascular relaxation was impaired by the addition of oxidized low-density cholesterol (LDL), but not affected by native LDL (35). Nonetheless, it cannot be ignored that oxidative stress causes atherosclerosis. It should be emphasized that, atherosclerosis per se, like inflammation, also causes oxidative stress (19,23).

In normotensive individuals and in those who are in the process of developing arterial hypertension, the action of NO synthesized in the endothelium is important to attenuate eventual hypertensive responses and vascular inflammatory conditions. Considering the vasodilatory and antiplatelet properties of NO, drugs capable of stimulating endogenous NO production, releasing NO exogenously, or acting via NO-related metabolites such as nitrite have been synthesized or identified. These drugs include dihydropyridine calcium channel blockers such as amlodipine, β-adrenergic antagonists such as carvedilol, organic nitrates such as glyceryl trinitrate, and 5-HT_2_ serotonergic antagonists such as ICI 169369 (36). However, NO acts as a double-edged sword, as high levels of NO can generate ONOO, a free radical with high chemical reactivity capable of causing or exacerbating oxidative stress, which contributes to the maintenance of hypertension (30,32,37).

In the search for drugs that can prevent atherosclerosis, emphasis has been on natural products that have been tested in animal models. For example, polyphenols found in olives such as hydroxytyrosol (HT) have been found to have antioxidant, anti-inflammatory, healing, and athero-protective properties (38). Recently, peptides obtained by hydrolysis of egg white have shown an effective effect in preventing or reversing oxidative stress (39,40). However, controlled, randomized, double-blind clinical trials for better definition are lacking.

## Oxidative stress induced by toxic metals: relationship with cardiovascular risk

**Figure 1:**
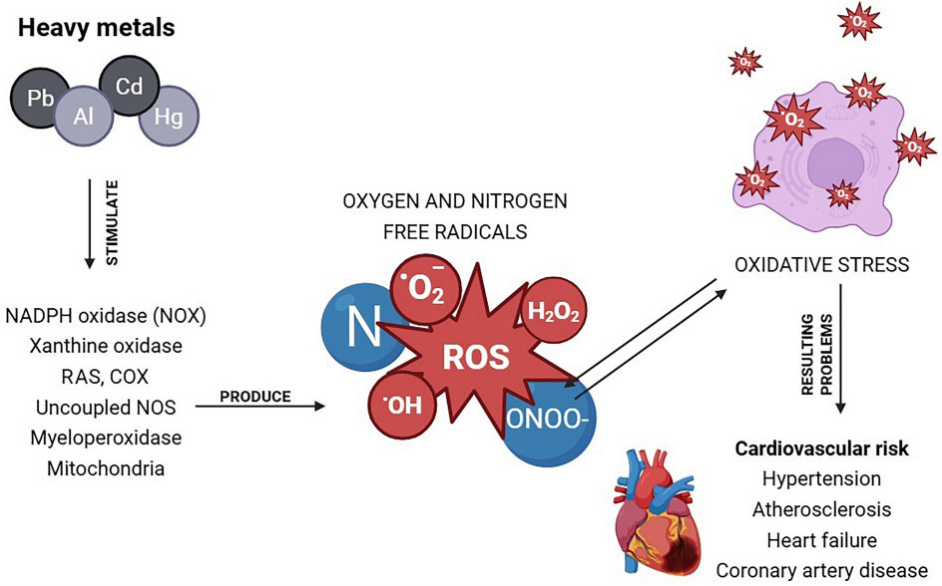
Schematic representation of reactive oxygen species (ROS) generation induced by heavy metals (Pb, Al, Cd, Hg), resulting in oxidative stress and leading to cardiovascular disorders. RAS: renin-angiotensin system.

Several “foreign” elements such as Hg, Al, Cd, and Pb can stimulate free radical production. Oxygen- and nitrogen-derived free radicals cause oxidative stress, which has deleterious effects on the organism (29,41). The high affinity of non-essentials metals for thiol groups appears to play a central role in their toxicity. Another common mechanism of oxidative stress activation by these metals involves the upregulation of NOX enzymes. This occurs through increased expression and activity, mediated by protein kinase C (PKC) activation, which subsequently enhances the activity of NOX1 and NOX4 isoforms. Additionally, these metals can stimulate the cyclooxygenase-2 (COX-2) isoform and, in some cases, promote oxidative stress through the activation of the Fenton reaction (10,11,36,41,42). This mechanism increases the production of ROS leading to oxidative stress and vascular dysfunction (Figure 1).

Low-level exposure to metals normally occurs from time to time. Metals are slowly deposited in tissues, such as bone, therefore intoxication is a silent and long-term process that only becomes harmful when deposits reach compromising levels. Consequently, observational and experimental studies have associated the exposure to toxic metals to risk of cardiovascular disease.

Hg, Al, Cd, and Pb are metals that are toxic to various organs and tissues in our body (9,10,11,29,43-61). Hg is absorbed and distributed in the form of vapor (pulmonary route), through the gastrointestinal tract (oral ingestion, soluble form), or through the skin and accumulates in almost all organs and tissues (3,44,62). The most commonly absorbed metal chemical forms are HgCl_2_, which is soluble in water, methyl mercury, which is absorbed in the digestive tract and accumulates in animals such as fish that are then consumed by humans, and metallic mercury (which generates vapors or aerosols) that can be absorbed due to its high solubility in lipids. After absorption, Hg is concentrated mainly in the kidneys and nervous tissue. Its serious and irreversible effects on the central nervous system are well known. Hg caustic action leads to tubular and glomerular injuries in the kidney and acute digestive disorders such as severe diarrhea due to damage to the intestinal mucosa, in addition to the toxic effects on other organs and tissues (chronic hydrargyrism).

Chronic Hg poisoning is the absorption of small amounts over prolonged periods of time, generally as a result of occupational exposure or food contamination. Once absorbed, Hg is distributed mainly to the central nervous system and kidneys, with long-term deposition in the bones. In unexposed populations, blood Hg concentrations are approximately 3 μg/L (∼11 nM), but in exposed individuals these values can reach 20 nM. Normally, high Hg levels generate ROS via NOX enzymes. However, a study found that in Hg-exposed infarcted animals, the mechanism of ROS generation is via xanthine oxidase (63). Interestingly, prior Hg exposure in rats with acute myocardial infarction, significantly increased mortality and correlated with a higher arrhythmia rate (64).

Humans and animals are exposed to Cd through a variety of routes (21,51,65). Human exposure occurs mainly through the consumption of contaminated food, active and passive inhalation of cigarette smoke, and occupational contamination of workers in the non-ferrous metals industry (66,67). Blood concentrations in workers exposed to Cd typically range from 5 to 50 µg/L, although extreme exposures can result in levels between 100 and 300 µg/L. Cd accumulates mainly in the kidneys, liver, and bones, with a biological half-life of 6 to 38 years in the kidneys and bones and 4 to 19 years in the liver (10).

The estimated Cd exposure levels in non-smoking adult men and women living in the United States are 0.35 and 0.30 μg Cd/kg per day, respectively. The tolerable weekly intake for Cd is 7 μg/kg body weight, which translates to 70 μg/day for a person weighing 70 kg. However, smoking exacerbates Cd exposure due to the natural accumulation of Cd in tobacco leaves (65). This metal is released along with other compounds in the combustion process of tobacco leaves. It is estimated that smokers are exposed to around 1.7 μg of Cd per cigarette, of which approximately 10% is inhaled during combustion. The average Cd blood level of smokers has been reported to be 1.58 μg/L, compared to the average American adult population estimate of 0.47 μg/L (51). After inhalation, the Cd present in cigarette smoke can be deposited in various tissues, including the aorta, with an average half-life of 10 to 30 years. Abu-Hayyeh et al. (66) demonstrated that the Cd content in the human aorta increases in direct proportion to the pack-years of cigarettes smoked, with selective accumulation in the media layer. The average Cd concentration accumulated in the middle layer of the human aorta was 7 µmol/L, which was sufficient to significantly reduce collagen synthesis in tissue cells. In our studies, acute Cd exposure affected vascular reactivity, promoting an increase in the contractile response to phenylephrine and endothelial dysfunction in aortic segments (58,59,68). The endothelial dysfunction observed in this experimental model is partially due to the reduction in NO bioavailability caused by the increased production of ROS from NOX enzymes. The increase in the contractile response to phenylephrine is associated with lower modulation of the endothelium and greater participation of O_2_^•-^, contractile prostanoids derived from COX-2 (PGE2 and TXA2), and the renin-angiotensin system (RAS) in this response (10).

Other toxic metals such as Pb and Al also promote increased oxidative stress as has already been described in various vessels (aorta, mesenteric, coronary) and in endothelial cells (52,60,69). A strong correlation with an increased risk of myocardial infarction and coronary heart disease has already been reported (45,70).

Population studies of the cardiovascular effects of Pb are focused on the association between exposure and arterial hypertension and the evidence is sufficient to infer a causal relationship between Pb exposure and high blood pressure (45,52,56,71). Thus, occupational exposure, air contamination, and contaminated drinking water and food are the main forms of Pb exposure. Once absorbed, Pb is estimated to remain in the blood for 30 days, in soft tissues for 40 days, and in bones for up to 27 years. Safe blood concentrations range from 2.4 to 16.6 µg/dL. However, acceptable levels in children are close to 5 µg/dL, due to the ability of Pb to easily cross the blood-brain barrier of the developing brain. However, according to the WHO, no level of Pb exposure is considered safe, as it is a non-essential and toxic metal. Pb blood concentrations between 20 and 44 µg/dL are enough to recommend a medical and environmental evaluation, while levels between 45 and 69 µg/dL are an indication for chelation therapy, and concentrations ≥70 µg/dL are considered a medical emergency (56,57).

Recent studies have demonstrated that Pb causes oxidative stress, mainly in the kidneys and cardiovascular tissues of animals (24,45,47,60). The increase in ROS may be related to the genesis and maintenance of hypertension by altering the balance of vasoconstricting and vasodilating factors, since the OH^•^ radical and other free radicals react with NO and reduce bioavailability (formation of ONOO-) of this endothelium-derived relaxing factor. Furthermore, OH^•^ binds to the -SH group of proteins, inducing arterial hypertension by altering other physiological mechanisms such as the activity of neurological and humoral components, causing sympathetic hyperactivity, baroreflex hyposensitivity, and reduction of vagal parasympathetic tone (69). Human studies on Pb show changes in neurotransmitters, suggesting increased activity of the sympathetic nervous system and of the renin-angiotensin system (72).

Al is the third most common and abundant toxic metal on Earth and often used in several industries and has potentially unlimited uses. It is present in food additives, antacids drugs, parenteral nutrition, and renal dialysis (55,73), as well as in water and tools used for food preparation. Consequently, foods and drinks containing Al are the main daily sources of exposure of this metal, being diet composition a key factor for the control of Al availability (46,55). Al can also be found in all tissues, with higher concentrations in the skeletal system and lungs. It is also deposited in the brain, liver, and muscles, leading to slow elimination from the body. In healthy individuals, Al serum concentration is approximately 10 µg/L but it can increase to up to 14 µg/L in exposed individuals. Toxic effects are observed when values exceed 100 µg/L (11).

Exposure to Al has been associated with prooxidant, mutagenic, and cytotoxic states (18,55), and Al exposure has been linked etiologically and epidemiologically to various neurological disorders such as Alzheimer's disease (50,74), Parkinson's disease (43), Guamanian-Parkinsonian complex, and amyotrophic lateral sclerosis (44).

Previous reports from animal experiments indicate that Al exposure at doses similar to human dietary levels impairs the cardiovascular system, increasing systolic blood pressure. Vasoconstrictor responses to phenylephrine, increased ROS production from NOX enzymes, and contractile prostanoids mainly from COX-2 were found in both the aorta and mesenteric arteries, while ACh-induced relaxation and NO bioavailability decreased (11,75). These effects were almost the same as Al exposure at much higher levels. Consequently, we must consider that frequent human dietary Al exposure might pose detrimental effects on the cardiovascular system, being an important public health risk.

Therefore, the established relationship between oxidative stress and cardiovascular disease raised the hypothesis that oxidative markers could be used to predict cardiovascular risk. Studies demonstrated that increased production of lipid peroxides may affect the role of lipoproteins in atherogenesis (76). Recently, among the possible biomarkers, malondialdehyde (MDA) level was reported to be one of the viable biomarkers of lipid oxidation in clinical trials (77). Authors have proposed the new biomarker growth differentiation factor 15 (GDF-15) that has predictive power in several scenarios involving cardiovascular disease (78). Other markers have also been studied, but larger prospective studies are required to explore whether baseline biomarkers of oxidative stress might contribute to cardiovascular risk prediction.

## Modulation of redox homeostasis: general mechanisms and perspectives for the therapy of cardiovascular disease

As oxidative stress is one of the main mechanisms related to cardiovascular disease, antioxidant pharmacological approaches are used to reduce oxidative stress. Drugs include ACE inhibitors and AT1 receptor antagonists, statins (inhibit HMG-CoA reductase), folic acid and 5-methylteahydrofolate (increases eNOS activity), polyphenolic antioxidants (decrease NOX enzymes activity), 5,7,8-tetrahydrobiopterin (BH4-eNOS cofactor), vitamin C and E, and peptides such as egg white hydrolysates (39,40).

The *in vivo* confirmation of the therapeutic efficacy of antioxidants in humans, however, has been challenging. In 1994, a large, randomized, double-blind clinical study (79) investigated the effects of vitamin E and beta carotene on the incidence of cancer in smokers. Neither antioxidant reduced cancer incidence in the sample, but rather increased mortality, raising the hypothesis that certain vitamin supplements may be more harmful than beneficial. A comprehensive meta-analysis has shown that treatments with beta carotene, vitamin A, and vitamin E can increase mortality (80). Later clinical work confirmed this trend. For example, vitamin C treatment did not reduce the risk of cancer in obese women (81). In addition, other large clinical studies revealed that vitamin E increased the risk of prostate cancer (82) and did not significantly alter the course of type 2 diabetes (83). Ristow et al. (84) observed a transient increase in free radicals with exercise with beneficial effects related to the prevention of insulin resistance in patients with type 2 diabetes mellitus and the use of antioxidants prevented these beneficial effects.

In addition, vitamins, natural products, and some synthetic compounds have different levels of antioxidant potential with possible therapeutic benefits. These include the ability to scavenge primary and secondary radicals, inhibit free-radical-induced membrane damage, and bind iron that can prevent radical formation. However, these bioactive compounds can also behave as prooxidants to facilitate ROS generation depending on certain conditions such as high pH, increased metal-ion (iron, copper, and zinc) transition, presence of oxygen or redox status, and depending on structural characteristics, concentration and cell type (85). This dual effect (antioxidant/prooxidant) makes it difficult to achieve therapeutic success with the use of these compounds. Critics of these mechanisms point out that there are no randomized, controlled trials showing the efficacy of antioxidant drugs in the treatment of arterial hypertension in humans. Thus, additional studies with other substances are needed to identify substances that combine therapeutic efficacy with low toxicity (26,19).

## Concluding remarks

Free radicals derived from O and N, as well as similar chemical species (especially H_2_O_2_ and NO), with pro-oxidant properties, are physiologically produced in our body, especially by mitochondria, through the respiratory chain. The human body has antioxidant protection mechanisms, whether enzymatic, such as SOD, or non-enzymatic, such as vitamins C and E. An imbalance between the oxidant chemical species and the endogenous antioxidant systems generates oxidative stress (1). Several lines of evidence suggest that oxidative stress participates in the pathophysiology of several cardiovascular diseases, in particular coronary atherosclerosis and its consequences (8,21,60). Unfortunately, we still do not have solutions for these diseases (86); however, the identification of biochemical mechanisms of “oxidative eustress” and “oxidative stress” may pave the way for further development of new solutions emphasizing the need to evaluate the effect of different antioxidants in the prevention of the damage caused by metal exposure on the cardiovascular system.

This review demonstrated that the toxic effects of metals are associated with a lower bioavailability of NO, greater production of ROS, greater participation of contractile prostanoids derived from COX, greater activity of the renin-angiotensin system, and increased vascular reactivity and are associated with the development of atherosclerosis. These effects reinforce the idea that exposure to toxic metals should be considered a risk factor for the development of cardiovascular diseases. The levels of such metals accepted as safe for humans by toxic surveillance agencies humans must be reduced.
